# Prediction of the wall-invasion pattern of advanced gallbladder carcinoma using extracellular volume fraction

**DOI:** 10.1007/s11604-025-01768-8

**Published:** 2025-03-19

**Authors:** Yukihisa Takayama, Takehiko Koga, Yoshihiro Hamada, Shinji Tanaka, Keisuke Sato, Ryo Murayama, Yusuke Ishida, Masatoshi Kajiwara, Kengo Yoshimitsu

**Affiliations:** 1 Department of Radiology, Faculty of Medicine, Fukuoka University, 7-45-1 Nanakuma, Jonan-ku, Fukuoka City, Fukuoka 814-0180 Japan; 2 Departments of Gastroenterology and Medicine, Faculty of Medicine, Fukuoka University, 7-45-1 Nanakuma, Jonan-ku, Fukuoka City, Fukuoka 814-0180 Japan; 3 Department of Pathology, Faculty of Medicine, Fukuoka University, 7-45-1 Nanakuma, Jonan-ku, Fukuoka City, Fukuoka 814-0180 Japan; 4 Department of Gastroenterological Surgery, Faculty of Medicine, Fukuoka University, 7-45-1 Nanakuma, Jonan-ku, Fukuoka City, Fukuoka 814-0180 Japan

**Keywords:** Gallbladder carcinoma, Wall-invasion type, Computed tomography, Extracellular volume fraction, Desmoplastic reaction

## Abstract

**Purpose:**

This study aimed to evaluate the utility of extracellular volume (ECV) fraction for predicting wall-invasion patterns in advanced gallbladder carcinoma (GBCA).

**Materials and methods:**

Patients who had surgically resected GBCA at a single institution were retrospectively evaluated. All patients underwent computed tomography (CT) before the surgery. Based on pathological examinations, the wall-invasion pattern of GBCA was classified into two groups: infiltrative growth (IG, n = 19) and destructive growth (DG, n = 11).

ECV map was generated by inputting the patients’ hematocrit values and subtraction algorithms using pre-contrast and equilibrium phase images. CT parameters were evaluated by two radiologists (Rad1 and Rad2). The Mann–Whitney *U* test was performed to identify significant CT parameters for differentiating between the two groups. The diagnostic ability was measured using receiver operating characteristic (ROC) curve analysis. Recurrence-free survival (RFS) was estimated using the Kaplan–Meier method, and differences between the two groups were compared using the log-rank test.

**Results:**

Thirty patients (mean age, 75.5 years; 20 men) were evaluated. Mean ECV fraction of the DG-type (Rad1, 34.5%; Rad2, 34.1%) was significantly higher than that of the IG-type (Rad1, 28.5%; Rad2, 28.8%) (p < 0.05). The ECV values of the two radiologists indicated that the areas under the ROC curves for differentiation between the two groups were Rad1, 0.91 and Rad2, 0.84 (p < 0.05). Medium RFS of the DG-type (970 days) was significantly shorter than that of the IG-type (2200 days) (p < 0.05).

**Conclusion:**

ECV fraction demonstrates potential as the most valuable predictor of the DG type of GBCA, which has a higher recurrence rate compared with the IG type. However, further large-scale multi-center studies are required to validate these findings.

## Introduction

Gallbladder carcinoma (GBCA) is one of the most common cancers in the world [1]. The 5-year survival rate in patients with GBCA with lymph node or distant metastasis is worse than those without metastasis [2]. Other poor prognostic factors of GBCA are age, tumor location, local spread, and vascular, lymphatic, and perineural invasions [3, 4]. Further, the wall-invasion pattern of advanced GBCA has been reported to be a poor prognostic factor [5].

The two types of wall-invasion patterns of advanced GBCA are (i) infiltrative growth (IG), in which cancer cells exhibit infiltrative growth in the muscle layer without muscle layer destruction; and (ii) destructive growth (DG), in which cancer cells exhibit massive growth with muscle layer destruction [5]. The DG-type exhibits aggressive growth, including a stromal desmoplastic reaction with fibroblasts and dense collagen fibers during tumor invasion, and has a poor prognosis compared with the IG-type [5]. The DG-type is also more frequently associated with other pathological findings that result in poor outcomes, such as poorly differentiated forms; lymphatic, venous and perineural invasions; higher Ki-67 levels; and lymph node and distant metastases [5–7].

Extracellular volume (ECV) fraction is the sum of the extracellular extravascular and intravascular spaces of tissues [8, 9]. Fibrosis occurs in the extracellular extravascular space but not in the intravascular space. The ECV fraction had a strong correlation with quantitatively assessed pathological fibrosis volume [10–16]. The ECV fraction is superior in predicting the degree of fibrosis compared with other computed tomography (CT) parameters, such as the CT value in the equilibrium phase or the absolute CT value [9, 14, 15]. Moreover, the ECV fraction is useful for predicting poor outcomes in carcinoma and pancreatic fistula [14, 15, 17, 18]. Koga et al. reported that the wall-invasion pattern can be predicted with high accuracy using apparent diffusion coefficient (ADC) [19]. The ECV fraction calculated from CT is considered superior to ADC for analyzing the wall-invasion pattern of GBCA because it is well-known that CT is superior to magnetic resonance imaging (MRI) in terms of spatial resolution [20–22]. However, no studies have yet utilized the ECV fraction to evaluate the wall-invasion pattern of GBCA. Therefore, we hypothesized that the ECV fraction could differentiate between DG- and IG-types by measuring the ECV fraction of the gallbladder wall where GBCA arose, and it might be useful to predict the difference in prognosis between the two different wall-invasion types of advanced GBCA. This study aimed to verify whether the ECV fraction is useful in predicting the wall-invasion patterns of advanced GBCA.

## Materials and methods

### Patients

**Fig. 1:**
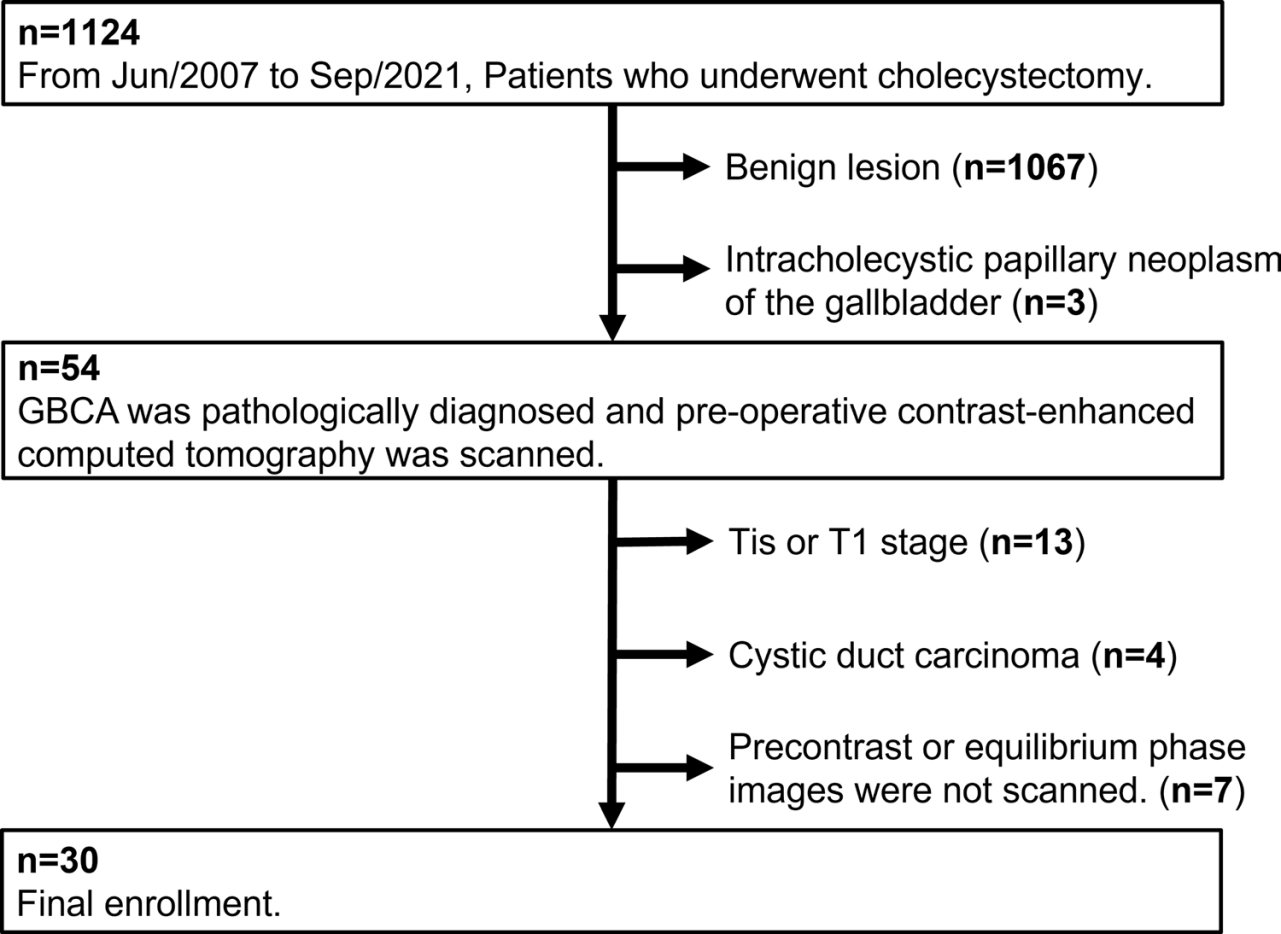
Patient selection flowchart

This study was approved by the Institutional Review Board of our institution. The requirement for written informed consent was waived because this study was a retrospective analysis of postprocessing CT images and clinical data. Between June 2007 and September 2021, 1124 consecutive patients who underwent cholecystectomy at our institution were investigated. The inclusion criteria were as follows: (1) pathological diagnosis of GBCA, and (2) preoperative contrast-enhanced CT examinations performed at our institution. The exclusion criteria were as follows: (1) benign lesion and intracholecystic papillary neoplasm, (2) GBCA without invasion of the muscular layer (Tis and T1 stages), (3) cystic duct carcinoma, and (4) if pre-contrast or equilibrium phase images were not scanned. A flowchart of the patient selection process is shown in Figure 1. Finally, 30 patients (19 IG-type and 11 DG-type) were enrolled in this study. The detailed patient characteristics are presented in Table 1. None of the enrolled patients underwent preoperative chemotherapy.

**Table 1:** Patient characteristics

Number of patients analyzed	30
Median age (range) years	75.5 (52–88)
Sex	
Female	10
Male	20
Wall-invasion type	
Destructive growth	11
Infiltrative growth	19

### CT protocols

The CT equipment used was an area- or multi-detector CT scanner (Aquilion ONE ViSION Edition or Aquilion 64; Canon Medical Systems, Tokyo). The scanning parameters were 0.5 mm × 80 row, 120 kVp, 3D auto-exposure control (Volume EC: SD12@5 mm), 0.5 s/rotation, 0.813 or 0.828 beam pitch, 512 × 512 matrix, 300–350 mm field of view, and 1- or 2-mm reconstruction. Among the 30 patients, nine underwent CT with noise reduction using a filtered back projection algorithm. The remaining 21 patients underwent CT with noise reduction achieved through hybrid iterative reconstruction (AIDR 3D; Canon Medical Systems). After obtaining pre-contrast images, 600 mgI/kg iodine contrast medium (Iopamiron 300 or 370, Bayer Health Care, Osaka) was injected for 30 s at a variable injection rate using the bolus tracking method and equilibrium phase images were obtained at 240 s after the commencement of contrast medium injection.

### ECV analysis

The ECV map was generated by inputting the hematocrit value (Ht) onto a workstation (Advanced Registration Software, Canon Medical Systems) specifically designed for this study. Subtraction between pre-contrast and equilibrium phase images was performed using SURE Subtraction Iodine Mapping (SSIM; Canon Medical Systems) [23, 24]. SSIM, designed for abdominal organs, employs a mutual information-based non-linear, non-rigid correction algorithm that is optimized for soft tissues. This enables precise alignment of volumetric data from non-contrast and multiphasic contrast-enhanced CT scans, facilitating the generation of iodine and ECV maps. The value of each voxel on the ECV map was multiplied by (100- Ht [%]). The Ht of each patient was obtained from blood samples taken within 1 week before and after the date of the CT examination.

Two radiologists (Rad1, Y.T. and Rad2, S.T.) with 24 and 10 years of experience in abdominal radiology, respectively, independently placed regions of interest (ROIs) on the images using a method for duplicating and positioning the areas on a workstation. The radiologists were blinded to the pathological results of the wall-invasion pattern; however, information regarding morphological features (e.g., flat or elevated) and tumor location (e.g., abdominal side or gallbladder bedside, tumor origin area) was provided by the coordinator.

**Fig. 2:**
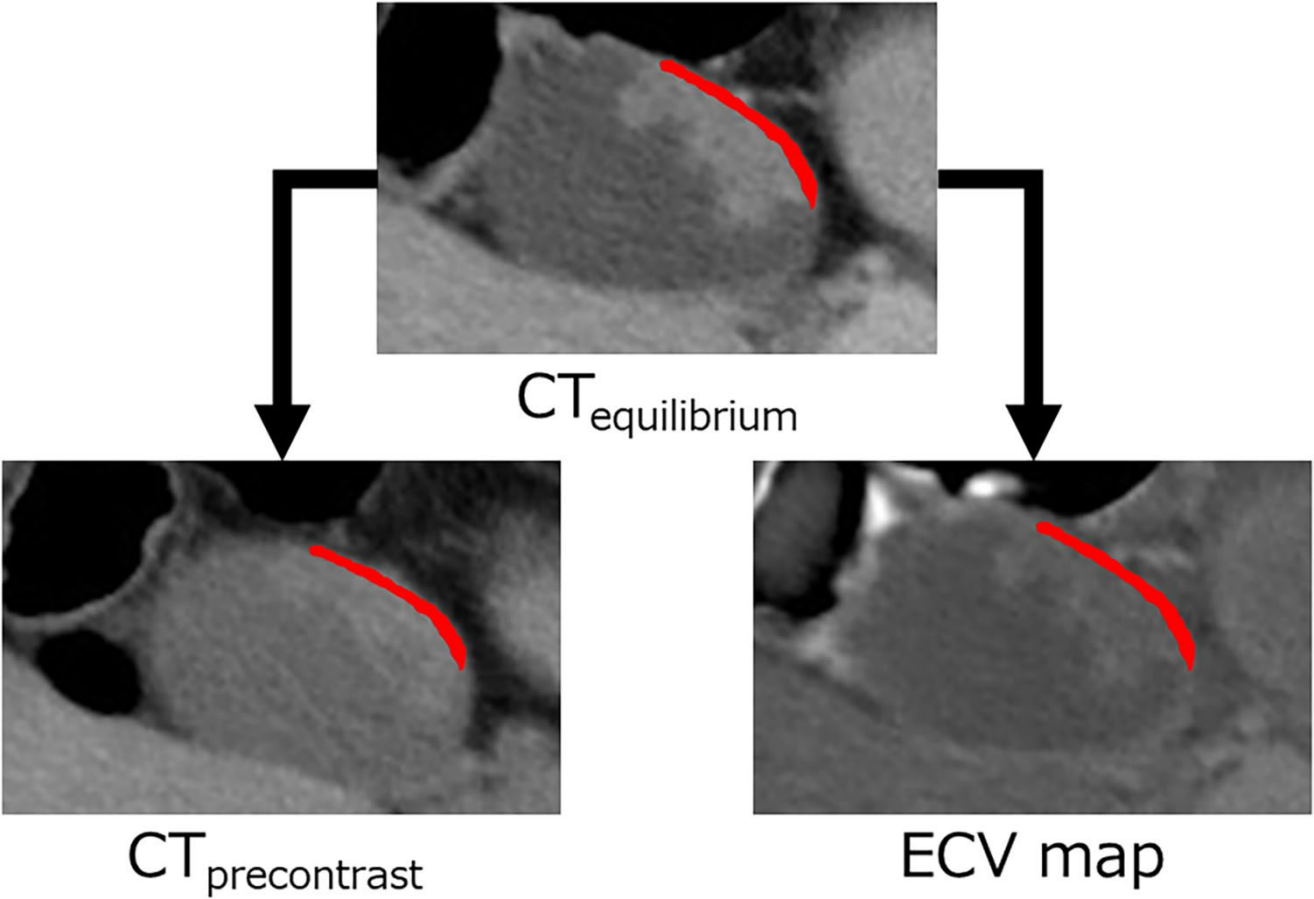
Images depicting duplicating and positioning of ROI on the gallbladder wall. The radiologists selected the maximal cross-section of GBCA on axial images, referencing equilibrium phase CT images (CT_equilibrium_), and delineated ROI on the wall where the GBCA is present, excluding perigallbladder fat and bile. The same ROIs are applied to the pre-contrast CT images (CT_precontrast_) and ECV map. Misalignments or ROIs extending outside the gallbladder wall are manually corrected. After correction, the ROIs are reviewed by the radiologist and coordinator to ensure proper positioning within the gallbladder wall. *ROI* region of interest, *GBCA* gallbladder carcinoma, *ECV* extracellular volume

Initially, the radiologists selected the maximal cross-section of the GBCA on axial images, referencing the equilibrium phase images, and delineated a sufficiently large ROI on the wall where the GBCA was present, carefully excluding perigallbladder fat and bile within the gallbladder. The same ROIs were duplicated and positioned on the pre-contrast images and ECV maps (Fig. 2). If misalignment occurred among the three image types, or if any ROI extended outside the gallbladder wall, manual corrections were performed. After correction, the ROIs were reviewed by the radiologist and the coordinator to confirm that the corrected ROIs were properly positioned within the wall where the GBCA had originated.

In addition to the mean ECV fraction, we calculated mean CT values of the gallbladder wall on pre-contrast and equilibrium phase images, hereafter referred to as “CT_precontrast_ and CT_equilibrium_ values, respectively,” and “ΔCT” was determined as a difference of mean CT values between pre-contrast and equilibrium phase images.

### Pathology and prognosis assessment

A study coordinator (T.K.), a gastroenterologist with 13 years of experience in the pancreatobiliary field, summarized all medical records and pathological results; however, he was blinded to the CT results. All the pathological features of surgical specimens were evaluated by the study coordinator and an experienced pathologist (Y.H.) with 25 years of experience in the pancreatobiliary field.

Gallbladder tissue samples for pathological examination were fixed with formalin, embedded in paraffin, sectioned, and stained with hematoxylin and eosin. The pathological findings were evaluated in terms of tumor size, operative method, macroscopic type, tumor location, histological differentiation, lymphatic invasion, venous invasion, perineural invasion, infiltration pattern, resection margin, and pathological TNM stage. The infiltration pattern into the surrounding tissues was evaluated based on the Japanese classification of biliary tract cancers: INFa, the tumor demonstrates expanding growth with a distinct border from the surrounding tissue; INFb, the tumor demonstrates an intermediate growth pattern between INFa and INFc; and INFc, the tumor demonstrates infiltrating growth with an indistinct border from the surrounding tissue [25]. The resection margins were examined and classified as R0, no residual tumor; R1, microscopic residual tumor; or R2, macroscopic residual tumor. Pathological stages were determined in accordance with the 8th edition of the Union for International Cancer Control TNM staging system [26]. In addition, the degree of fibrosis in the gallbladder wall between the muscularis propria and serosa at the tumor location was diagnosed by a pathologist (Y.H.) as mild, <33% fibrosis; moderate, 33–66% fibrosis; and severe, >66% fibrosis based on literature that was modified for this study [27]. For prognostic evaluation, the number of recurrence-free survival (RFS; days) was determined as the time from the date of surgery to recurrence or last follow-up examination. Overall survival (OS) was defined as the time from the date of surgery to death or last follow-up examination.

### Statistical analyses

The duration from the CT examination to surgery, and CT parameters between the DG- and IG-type were compared using the Mann–Whitney *U* test. We calculated the intraclass correlation coefficient (ICC) using a two-way random-effects model to describe the correlations of the measurement of CT parameters between the two radiologists based on the following criteria: <0.40, poor agreement; 0.40–0.59, fair agreement; 0.60–0.74, good agreement; and ≥0.75, excellent agreement.

Nonparametric receiver operating characteristic (ROC) analysis was performed to investigate the diagnostic performance of each imaging parameter. The sensitivity and specificity of the CT parameters were compared pairwise using McNemar's test. The areas under the curves (AUCs) of the CT parameters were compared non-parametrically using the ROCPLOT macro (SAS Corporation, Cary, NC). First, a multiple comparison of the AUCs was conducted among the CT parameters. If a statistically significant difference was identified, pairwise comparisons of their AUCs were subsequently performed.

Pathological findings of GBCA between the two groups were compared using the chi-square test or Mann–Whitney *U* test. Comparisons of the ECV fraction among the three degrees of fibrosis in the gallbladder wall at the tumor location were analyzed without separating the DG- and IG-type. They were compared using the Kruskal–Wallis test and Bonferroni post hoc test for pairwise comparisons. Spearman's rank correlation coefficient (Spearman's ρ) was also calculated. Estimation and comparison of RFS and OS between the two groups were performed using the Kaplan–Meier log-rank test. Statistical significance was set at *p* < 0.05 (*p*< 0.0167 was used for the Bonferroni post-hoc test) All statistical analyses were performed using the JMP Pro14.0.0 (SAS Corporation, Cary, NC).

## Results

### Comparisons of CT parameters

The mean and standard deviation (SD) of the duration between CT examination and surgery were 26.6 ± 16.9 days for the DG-type and 25.8 ± 20.4 days for the IG-type. There was no significant difference between the two groups (*p*=0.80). The mean and SD of the ROI area were 27.5 ± 12.1 mm^2^ for R1 and 22.7 ± 7.3 mm^2^ for R2.

The results of the comparisons of CT parameters between the DG and IG types are shown in Table 2. Both radiologists found significant differences in the ECV fraction between the two groups (*p*<0.05). Regarding CT_equilibrium_ and ΔCT, one radiologist (Rad1) found significant differences between the two groups (*p*<0.05), while the other radiologist did not (*p*>0.05). None of the radiologists found significant differences in CT_precontrast_ between the two groups (*p*>0.05).

**Table 2:** Results of comparisons of CT parameters and diagnostic ability

	Radiologist	Mean ± SD		Mann–whitney *U* test	ROC analysis				ICC
		DG	IG	p value	Sensitivity [%]	Specificity [%]	AUC (95%CI)	Cut off value	
CT value [H.U.] (pre-contrast)	R1	34.8 ± 7.8	31.1 ± 7.0	0.20	81.8	52.6	0.65 (0.41–0.83)	31.5	0.57
	R2	36.1 ± 5.9	34.8 ± 5.6	0.62	36.4	84.2	0.55 (0.33–0.76)	39.5	
CT value [H.U.] (equilibrium phase)	R1	101.6 ± 9.0	87.8 ± 14.1	0.004*	81.8	78.9⁑	0.82 (0.61–0.93)	96.5	0.88
	R2	98.6 ± 12.6	89.6 ± 12.7	0.08	90.9	57.9	0.70 (0.46–0.86)	91.1	
ΔCT value [H.U.]	R1	66.8 ± 7.4	57.6 ± 13.0	0.048*	100.0	52.6	0.72 (0.51–0.87)	57.8	0.79
	R2	62.5 ± 15.0	54.8 ± 10.7	0.07	81.8	63.2	0.70 (0.47–0.87)	57.8	
ECV fraction [%]	R1	34.5 ± 2.6	28.5 ± 3.2	<0.001*	100.0	84.2†	0.91 (0.72–0.97)†	30.3	0.92
	R2	34.1 ± 4.1	28.8 ± 3.8	0.003*	72.7	78.9	0.84 (0.65–0.93)	32.0	

The ICC is calculated to describe correlations of the CT measurements between the two radiologists, based on the following criteria: <0.40, poor agreement; 0.40–0.59, fair agreement; 0.60–0.74, good agreement; and ≥0.75, excellent agreement

*SD* standard deviation, *DG* destructive growth, *IC* infiltrative growth, *ROC* receiver operating characteristic, *AUC* area under the curve, *CI* confidence interval, *ICC* intraclass correlation coefficient

^*^Indicates significant difference between DG- and IG-types (p< 0.05)

⁑Indicates significant difference with CT value (pre-contrast) (p< 0.05)

^†^Indicates significant difference with CT value (pre-contrast) and ΔCT value (p< 0.05)

The inter-reader agreement between the two radiologists is shown in Table 2. Although CT_precontrast_ showed fair agreement, CT_equilibrium_, ΔCT and the ECV fraction showed excellent and significant agreements (*p*<0.05).

### Diagnostic ability of CT parameters

The diagnostic performance for differentiating DG-type from IG-type, as analyzed using ROC curves, is summarized in Table 2. In the pairwise comparison of sensitivity, specificity, and AUC, Rad1 demonstrated that the specificity of CT_equilibrium_ was significantly higher than that of CT_precontrast_ (*p*<0.05). Additionally, the specificity and AUC of the ECV fraction were significantly higher than those of CT_precontrast_ and ΔCT (*p*<0.05). In contrast, no significant differences in sensitivity were observed among the CT parameters (*p*>0.05). Rad2 demonstrated that there were no significant differences in any pairwise comparison of sensitivity, specificity, or AUC among the CT parameters (*p*>0.05).

### Comparisons of pathological assessment

Comparisons of the pathological findings between the DG- and IG-types are presented in Table 3. There was a significant difference in tumor location between the two groups (*p*<0.05). The incidences of venous and perineural invasion were significantly higher in the DG-type than in the IG-type (*p*<0.05). In addition, the degree of fibrosis in the gallbladder wall at the tumor location of the DG-type was significantly higher than that of the IG-type (*p*<0.05). Other pathological findings were not significantly different between the two groups (*p*>0.05).

**Table 3:** Comparison of pathological findings

		DG	IG	p value
Tumor size	Mean diameter (mm)	38.8	30.0	0.31
Operative method	Extended cholecystectomy	4	9	0.34
	Simple cholecystectomy	7	10	
Macroscopic type	Flat	2	3	0.26
	Nodular	9	12	
	Papillary	0	4	
Tumor location	Neck	5	1	0.03*
	Body	0	6	
	Fundus	4	7	
	Diffuse	2	5	
Histological differentiation	Well-differentiated	7	14	0.40
	Moderately differentiated	3	5	
	Poorly differentiated	1	0	
Lymphatic invasion	Positive	11	15	0.10
	Negative	0	4	
Venous invasion	Positive	11	13	0.04*
	Negative	0	6	
Perineural invasion	Positive	11	12	0.02*
	Negative	0	7	
T stage	T2	9	17	0.41
	T3	2	1	
	T4	0	1	
N stage	N0	4	4	0.36
	N1	7	15	
	N2	0	0	
M stage	M0	11	18	0.44
	M1	0	1	
Infiltration pattern	INFa	0	0	0.24
	INFb	10	17	
	INFc	1	0	
	Unclassifiable	0	2	
Final pathological stage	II	7	13	0.91
	III	3	5	
	IV	1	1	
Resection margin	R0	10	15	0.52
	R1	1	2	
	R2	0	2	
Degree of fibrosis in the GB wall	Mild	1	8	0.03*
	Moderate	2	6	
	Severe	8	5	

*GB* gallbladder, *DG* destructive growth, *IG* infiltrative growth

**Fig. 3:**
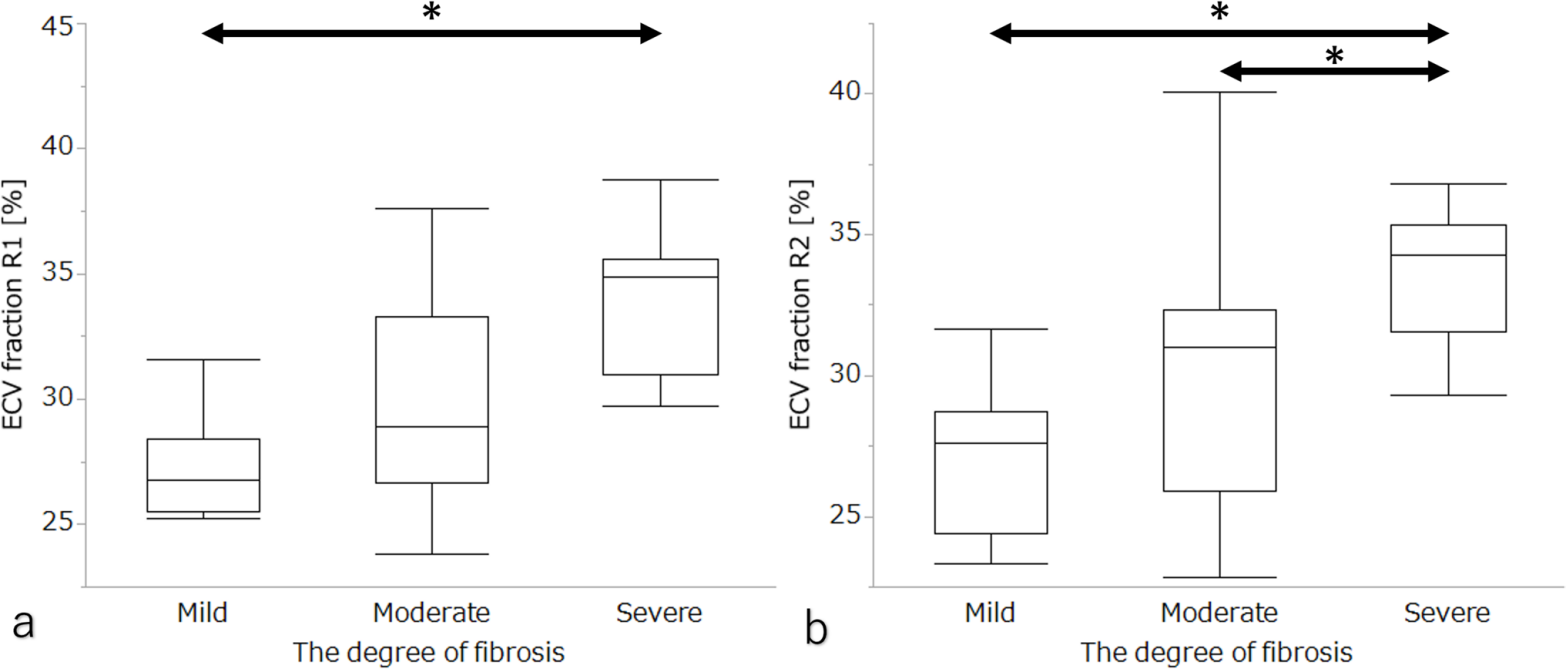
Box-plot graphs of ECV fraction for each fibrosis grade of the gall bladder wall is calculated by two radiologists. Comparison of the ECV fraction among three degrees of fibrosis in the gallbladder wall at tumor location are analyzed without separating DG- and IG-type. * indicates a significant difference identified by the Bonferroni post-hoc test (p < 0.0167). The mean and standard deviations of ECV fraction from two radiologists for mild, moderate and severe degrees of fibrosis in the gallbladder wall are as follows: R1: 27.3±1.0, 29.4±1.1, and 33.7±0.8; and R2: 27.5±1.3, 29.5±1.3, and 33.9±1.1, respectively. *ECV* extracellular volume, *DG* destructive growth, *IG* infiltrative growth

Regarding the relationship between the ECV fraction and the degree of fibrosis in the gallbladder wall at the tumor location, both radiologists indicated that the ECV fraction had a significantly positive correlation with the degree of fibrosis in the gallbladder wall (*p*<0.05). The Spearman’s ρ of two radiologists were: Rad1, 0.73 and R2, 0.66. In addition, Rad1 showed significant differences in the ECV fractions between the mild and severe groups (Fig. 3a; *p*<0.0167), and Rad2 showed significant differences between the mild and severe groups, and between the moderate and severe groups (Fig. 3b; *p*<0.0167).

### Comparison of RFS and OS

Two patients with the IG type were excluded from the comparison of RFS and OS because they died of gastric carcinoma and postoperative death. Ten of the 11 patients (90.9%) with the DG type and 10 of the 17 patients (58.8%) with the IG type received postoperative adjuvant chemotherapy; however, there was no significant difference between the two groups postoperative follow-up period (*p*<0.05).

The medium postoperative follow-up period was 1569 days. During the follow-up period, GBCA recurrence was observed in five of 11 patients (45.5%) with the DG type and seven of 17 patients (41.2%) with the IG type. Among the five recurrent patients in the DG type, one experienced local recurrence, and four developed distant metastases. All the five patients were classified as R0. Four patients received postoperative S1 chemotherapy, whereas one did not and subsequently developed distant metastasis.

Among the seven recurrent patients in the IG type, five experienced local recurrences, and two developed distant metastases. Of these, four patients were classified as R0; however, three developed local recurrences, and one developed distant metastasis. One patient received gemcitabine chemotherapy, another received S1 chemotherapy, and two did not undergo any chemotherapy. All four showed no recurrence. Two patients were classified as R1—one received postoperative S1 chemotherapy but developed local recurrence, whereas the other did not receive chemotherapy and developed distant metastasis. Additionally, one patient was classified as R2 and received postoperative gemcitabine chemotherapy, but still developed local recurrence.

**Fig. 4:**
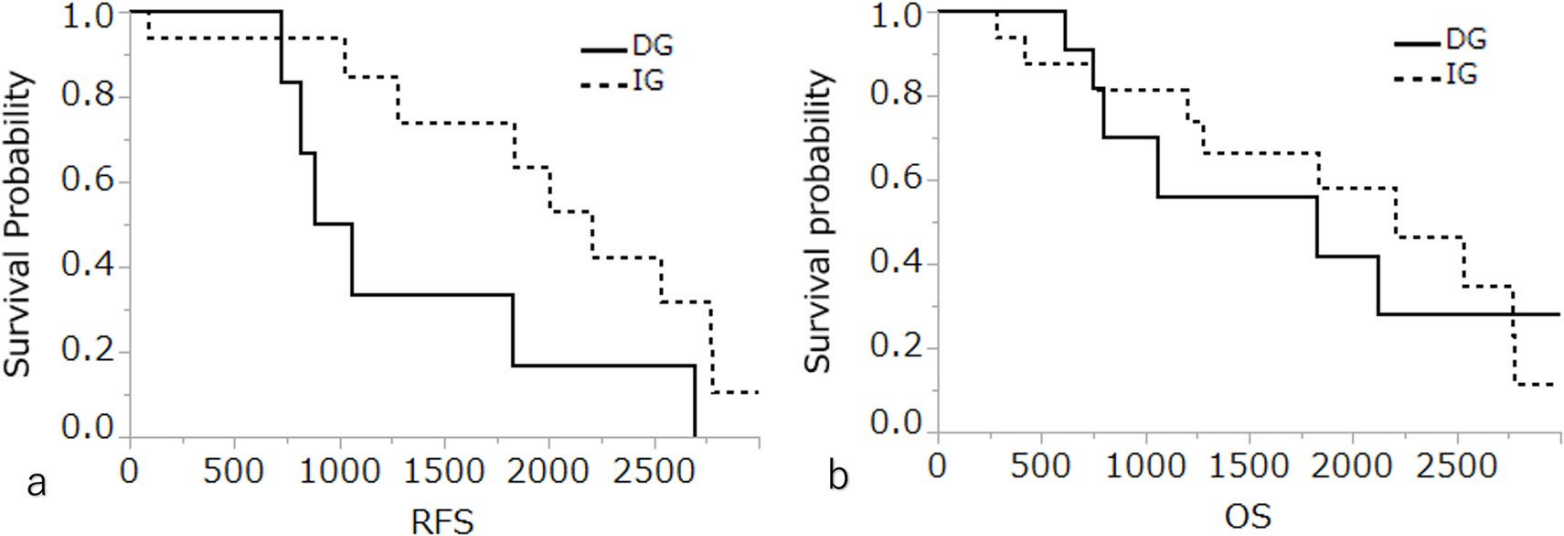
Kaplan–Meier curve for a) recurrence-free survival (RFS) and b) overall survival (OS) according to the wall-invasion pattern of gall bladder carcinoma. The medium RFS of the destructive growth (DG) type (970 days) is significantly shorter than that of the infiltrative IG-type (2200 days) (p < 0.05). The medium OS of DG- and IG-types are 1828 and 2200 days, but is was no significant difference in OS between the two groups (p > 0.05). *DG* destructive growth, *IG* infiltrative growth

Medium RFS of the DG-type (970 days) was significantly shorter than that of the IG-type (2200 days) (Fig. 4a; *p*<0.05). Death related to GBCA was observed in four of 11 patients (36.4%) and in five of 17 patients (29.4%) with the DG- and IG-types, respectively. There was no significant difference in OS between the two groups (Fig. 4b; *p*>0.05).

**Fig. 5:**
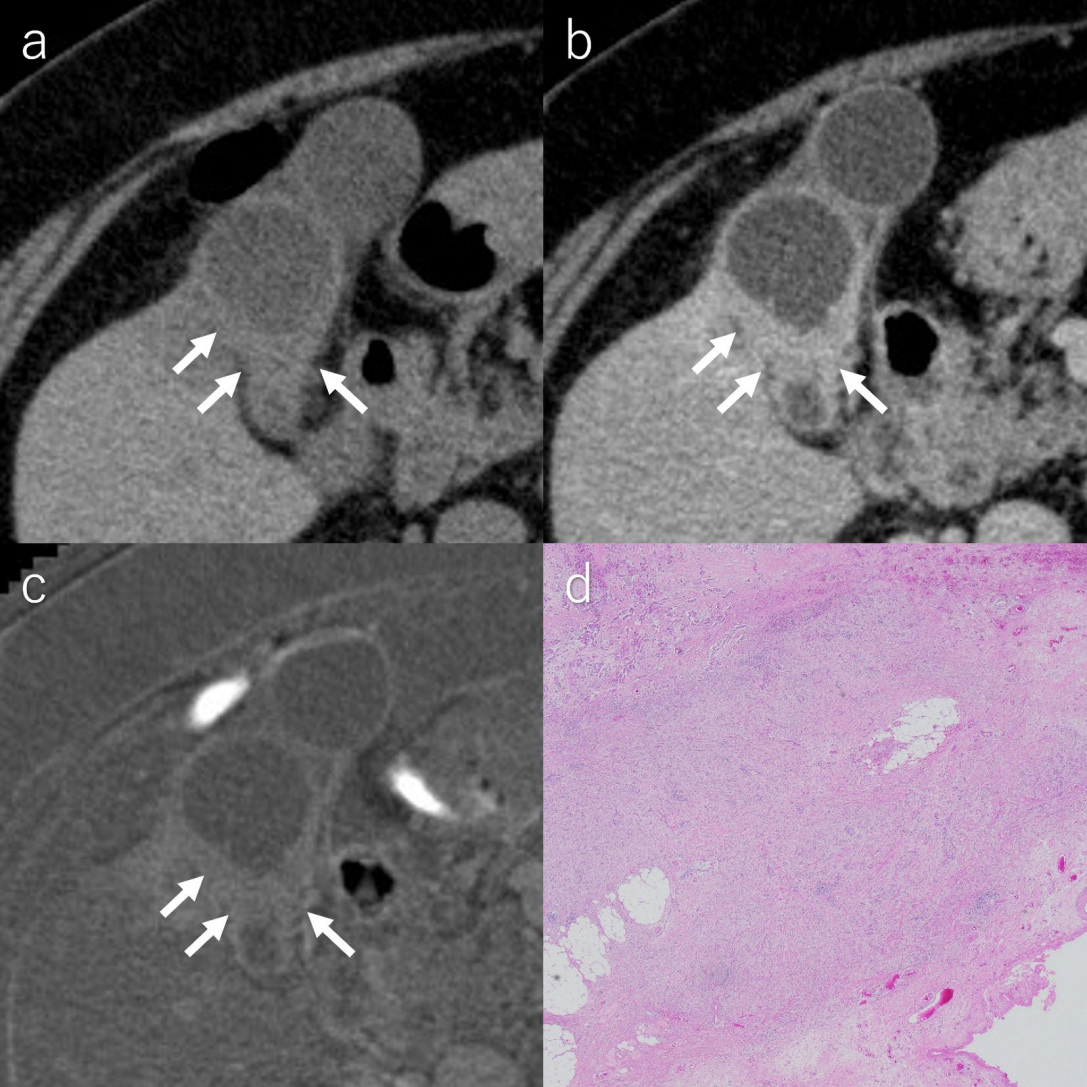
A 65-year-old female with the DG-type of gallbladder carcinoma. **a** Pre-contrast CT image, **b** equilibrium phase CT image and **c** hematoxylin and eosin-stained microscopic images. The ECV fractions calculated by two radiologists are 35.7% and 36.8%. The degree of fibrosis in the gallbladder wall is pathologically diagnosed as severe grade. *CT* computed tomography, *DG* destructive growth, *ECV* extracellular volume

**Fig. 6:**
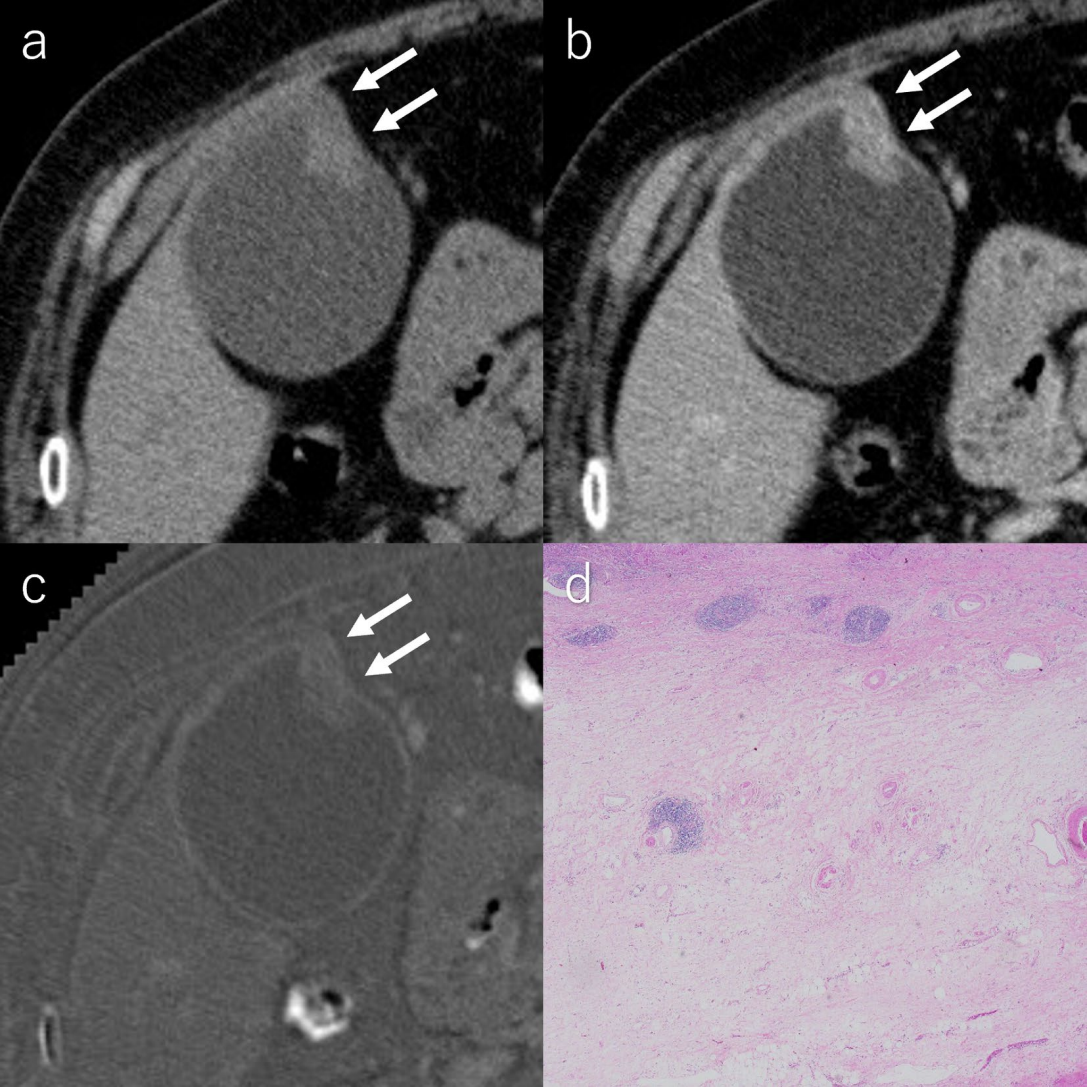
A 72-year-old female with the IG-type of gallbladder carcinoma. **a** Pre-contrast CT image, **b** equilibrium phase CT image and **c** hematoxylin and eosin-stained microscopic images. The ECV fractions calculated by two radiologists are 25.8% and 23.4%. The degree of fibrosis in the gallbladder wall is pathologically diagnosed as mild grade. *ECV* extracellular volume, *CT* computed tomography, *IG* infiltrative growth

Representative images of the three imaging techniques of DG- and IG-types are depicted in Figures 5 and 6, respectively.

## Discussion

Our study revealed that the ECV fraction of the DG-type was significantly higher than that of the IG-type. Both the radiologists revealed that the ECV fraction might be a useful predictor of DG type compared with other CT parameters, as determined by the Mann–Whitney *U* test. The ECV fraction significantly correlated with the degree of fibrosis in the gallbladder wall at the tumor location. In addition, prediction of the DG-type using the ECV fraction would be useful for estimating the likelihood of tumor recurrence.

The DG-type exhibited a significantly higher ECV fraction than the IG-type. Compared with pathological examinations, the ECV fraction showed a significant positive correlation with the degree of fibrosis in the gallbladder wall at the tumor location, with an excellent inter-reader agreement. In addition, fibrosis in the gallbladder wall is associated with desmoplastic reactions during tumor invasion. A more severe fibrosis grade was observed in the DG-type than in the IG-type. Although this is the first study on the ECV fraction of GBCA, our results are consistent with previous reports that evaluated the degree of fibrosis in other tissues using the ECV fraction [10–12, 14, 23].

In differentiating between DG- and IG-types, both the ECV fraction and CT_equilibrium_ demonstrated good diagnostic performance. While the ECV fraction had a higher AUC than CT_equilibrium_ for both radiologists, the difference was not statistically significant, possibly due to the limited number of patients analyzed. Both CT parameters increase with the concentration of iodine contrast agents in the extracellular space (ECS) and are associated with the degree of fibrosis in this space [5, 10–18, 23, 24]. Previous studies have also demonstrated that the ECV fraction is more sensitive to the degree of fibrosis in the ECS compared with CT_equilibrium_ [9, 14, 15]. In this study, the ECV fraction was calculated by normalizing changes in tumor or tissue CT values before contrast administration and at the equilibrium phase using aortic CT value changes [8, 28]. This reduces variability owing to differences in body size, renal function, and image noise. At equilibrium, the contrast agent is distributed equally between the ECS and blood. However, it remains only in the plasma (100 - Ht [%]) and does not enter the red blood cells [8, 29, 30]. Therefore, this correction is applied to accurately estimate the plasma volume. These corrections enhance the accuracy and consistency of the ECV fraction in assessing ECS volume and fibrosis compared with CT_equilibrium_ or ΔCT.

Our study suggests that it is important to differentiate between the two groups using the ECV fraction to predict patient prognosis. The DG-type group showed a significantly shorter RFS than the IG-type group, although there was no significant difference in patients who received postoperative adjuvant chemotherapy between the two groups. These results may be related to the poor prognostic pathological factors of the DG-type [3–7]. However, there was no significant difference in the OS between the two groups, which may be due to the effects of chemotherapy after recurrence. In patients with advanced GBCA at T3 or T4 stage, it is difficult to examine the detailed pathological factors using needle biopsy. For such patients, the ECV fraction may provide information for estimating tumor aggressiveness, especially for predicting the likelihood of tumor recurrence. Unfortunately, owing to the limited number of cases analyzed, it is difficult to discuss the need for changes in surgical procedures or the necessity for preoperative chemotherapy based on our results, in relation to DG- and IG-types.

The degree of fibrosis in the gallbladder wall is not a poor prognostic factor, unlike other bile tract carcinomas, such as intrahepatic cholangiocarcinoma and bile duct carcinoma [3, 31–33]. One of the speculated reasons for this is that fibrosis is also accompanied by inflammation, such as cholecystitis. In the present study, we confirmed that fibrosis was not caused by inflammation in any of the enrolled subjects. We investigated whether the ECV fraction, divided into two groups based on cutoff values determined by ROC analysis, could predict prognosis for RFS and OS. However, no significant results were obtained (data not shown). Therefore, we presumed that the ECV fraction was not directly related to the prediction of GBCA prognosis but was helpful in estimating the likelihood of GBCA recurrence by predicting the wall-invasion pattern.

In this study, an overlap in ECV fractions was observed between the two groups. Although a statistically significant difference was identified and the ROC curve suggested that the ECV has the potential for good diagnostic ability, it may still be challenging to strictly differentiate between the two groups. Notably, the DG-type tended to have a higher ECV fraction in the enrolled patients. In addition, the IG-type showed lower to equivalent ECV fractions than the DG-types. If the ECV fraction in the gallbladder wall at the tumor location was low (e.g., < 30%), there was a high probability of the IG-type. Conversely, if the ECV fraction in the gallbladder wall at the tumor location was high (e.g., > 30%), there was a possibility of either the DG- or IG-types; however, the DG-type was suspected more. However, further studies are necessary to evaluate whether our hypotheses are correct.

A previous study examined whether the ADC could be used to differentiate between DG- and IG-types and concluded that the wall-invasion pattern of advanced GBCA can be predicted by ADC [19]. Although the diagnostic ability of the T-stage of GBCA between CT and MRI was equivalent [20, 34, 35], there are no studies regarding the diagnostic ability to differentiate between DG- and IG-types by comparing the ECV fraction and ADC. In this study, we were unable to determine whether the ECV fraction was superior or inferior to ADC. This limitation should be acknowledged as a potential drawback of the study.

This study had other limitations. First, we analyzed a small number of patients from a single center, which limited our ability to avoid speculation and bias. The retrospective design of this study is also a potential source of bias. Thus, these findings may require validation in a large-scale, multicenter study. Second, in this study, we calculated the ECV fractions from the pre-contrast and equilibrium phase images, so we could not avoid misregistration between the two-phase images, even though we applied SURE algorithms. Although we drew ROIs avoiding perigallbladder fat and bile near the gallbladder, we might have inevitably miscalculated the ECV fraction of the gallbladder wall because of the small size of the ROIs. A potential solution to address this misregistration issue is the use of dual-energy CT, which enables the simultaneous acquisition of data from the same slice of the gallbladder using two X-ray sources or detectors [36–38]. Further, the gallbladder has a pear-shaped morphology with thin walls. We evaluated the CT parameters of the GBCA on axial images using thin-slice thickness. However, we could not apply multiplanar reconstruction technique (MPR) to ECV maps because of technical difficulties. MPR images may be helpful in pursuing more accurate evaluations.

## Conclusion

CT parameters, except for CT_precontrast_, may have the potential to predict the DG type of GBCA, which exhibits greater tumor aggressiveness and a higher likelihood of recurrence than the IG type. In particular, ECV fraction seems to be the most valuable indicator for distinguishing between the two groups of GBCA. However, further large-scale, multi-center studies are required to validate these findings.

## Declarations

### Generative AI in scientific writing

The authors of this manuscript did not use any generative AI and AI-assisted technologies in scientific writing.
