# Correction to: Prediction of the wall‑invasion pattern of advanced gallbladder carcinoma using extracellular volume fraction

**DOI:** 10.1007/s11604-025-01868-5

**Published:** 2025-09-15

**Authors:** Yukihisa Takayama, Takehiko Koga, Yoshihiro Hamada, Shinji Tanaka, Keisuke Sato, Ryo Murayama, Yusuke Ishida, Masatoshi Kajiwara, Kengo Yoshimitsu

**Affiliations:** 1 Department of Radiology, Faculty of Medicine, Fukuoka University, 7-45-1 Nanakuma, Jonan-ku, Fukuoka City, Fukuoka 814-0180 Japan; 2 Departments of Gastroenterology and Medicine, Faculty of Medicine, Fukuoka University, 7-45-1 Nanakuma, Jonan-ku, Fukuoka City, Fukuoka 814-0180 Japan; 3 Department of Pathology, Faculty of Medicine, Fukuoka University, 7-45-1 Nanakuma, Jonan-ku, Fukuoka City, Fukuoka 814-0180 Japan; 4 Department of Gastroenterological Surgery, Faculty of Medicine, Fukuoka University, 7-45-1 Nanakuma, Jonan-ku, Fukuoka City, Fukuoka 814-0180 Japan

**Correction: Japanese Journal of Radiology** 10.1007/s11604-025-01768-8

An error was identified in the *Patients* section of the Materials and Methods and in Fig. 1 of the above-mentioned article.

The correct description is as follows:

**Patients**

Between January 2009 and September 2021, 1,124 consecutive patients who underwent cholecystectomy at our institution were investigated, instead of “Between June 2007 and September 2021.”

